# Ultrasensitive Quantification of Multiple Estrogens in Songbird Blood and Microdissected Brain by LC-MS/MS

**DOI:** 10.1523/ENEURO.0037-22.2022

**Published:** 2022-07-14

**Authors:** Cecilia Jalabert, Maria A. Shock, Chunqi Ma, Taylor J. Bootsma, Megan Q. Liu, Kiran K. Soma

**Affiliations:** 1 Department of Zoology, University of British Columbia, Vancouver, British Columbia, Canada V6T 2B5; 2 Djavad Mowafaghian Centre for Brain Health, University of British Columbia, Vancouver, British Columbia, Canada V6T 2B5; 3 Department of Psychology, University of British Columbia, Vancouver, British Columbia, Canada V6T 2B5; 4 Graduate Program in Neuroscience, University of British Columbia, Vancouver, British Columbia, Canada V6T 2B5

**Keywords:** aromatase, estrogen profiling, mass spectrometry, microdissected brain, social behavior network, songbird

## Abstract

Neuroestrogens are synthesized within the brain and regulate social behavior, learning and memory, and cognition. In song sparrows, *Melospiza melodia*, 17β-estradiol (17β-E_2_) promotes aggressive behavior, including during the nonbreeding season when circulating steroid levels are low. Estrogens are challenging to measure because they are present at very low levels, and current techniques often lack the sensitivity required. Furthermore, current methods often focus on 17β-E_2_ and disregard other estrogens. Here, we developed and validated a method to measure four estrogens [estrone (E_1_), 17β-E_2_, 17α-estradiol (17α-E_2_), estriol (E_3_)] simultaneously in microdissected songbird brain, with high specificity, sensitivity, accuracy, and precision. We used liquid chromatography tandem mass spectrometry (LC-MS/MS), and to improve sensitivity, we derivatized estrogens using 1,2-dimethylimidazole-5-sulfonyl-chloride (DMIS). The straightforward protocol improved sensitivity by 10-fold for some analytes. There is substantial regional variation in neuroestrogen levels in brain areas that regulate social behavior in male song sparrows. For example, the auditory area NCM, which has high aromatase levels, has the highest E_1_ and 17β-E_2_ levels. In contrast, estrogen levels in blood are very low. Estrogen levels in both brain and circulation are lower in the nonbreeding season than in the breeding season. This technique will be useful for estrogen measurement in songbirds and potentially other animal models.

## Significance Statement

Estrogens are locally synthesized within the brain and play important roles in neural function, but measurement of estrogens in specific brain regions is extremely challenging. We developed a method using liquid chromatography tandem mass spectrometry (LC-MS/MS) for the measurement of estrone (E_1_), 17β-estradiol (17β-E_2_), 17α-estradiol (17α-E_2_), and estriol (E_3_) in microdissected brain, plasma, and blood in a songbird model. There are robust regional differences and seasonal changes in estrogen levels. This technique will be useful for measurement of estrogens and will facilitate studies of neuroestrogens and their functions.

## Introduction

The brain can locally produce steroids, either *de novo* from cholesterol or from conversion of circulating precursors (Schlinger et al., 1999; Schmidt et al., 2008). The brain expresses all the necessary enzymes for steroid synthesis (Tsutsui, 2011) in a region-specific manner (Soma et al., 2003; Wacker et al., 2010). Brain-derived steroids, known as neurosteroids, were first characterized in rodents (Baulieu, 1991; Mellon et al., 2001; Hojo and Kawato, 2018), and later in other vertebrates such as birds (Schmidt et al., 2008; Tsutsui, 2011; Schlinger, 2015). In particular, estrogens are produced from androgens by the aromatase enzyme, which exhibits high activity in specific brain regions (Naftolin and Ryan, 1975). Bioactive estrogenic metabolites such as catechol estrogens are also locally synthesized within the brain, although their functions are less clear (Fowke et al., 2003; Denver et al., 2019b).

High neuroestrogen production occurs in songbirds, and seasonal changes in local estrogen production affect aggressive behavior. The song sparrow, *Melospiza melodia*, which is common along the Pacific Coast of North America, is an excellent model for investigating neurosteroid production and the regulation of territorial aggression (Patten and Pruett, 2009; Wacker et al., 2010). Males exhibit aggression during breeding (spring) and nonbreeding (autumn) seasons but not during molt (late summer; Wingfield and Hahn, 1994; Soma et al., 2003). Circulating levels of testosterone are high during the breeding season but very low during the nonbreeding season. In the nonbreeding season, castration does not reduce aggression, but inhibition of aromatase does reduce aggression (Wingfield, 1994; Soma et al., 2000a, b). Administration of 17β-estradiol (17β-E_2_) increases aggression in nonbreeding males (Heimovics et al., 2015). Social behavior, including aggression, is regulated by the social behavior network (SBN). The SBN expresses steroidogenic enzymes as well as sex steroid receptors, indicating that this circuit is both steroid-synthetic and steroid-sensitive (Newman, 1999; Goodson, 2005). In song sparrows, aromatase is highly expressed in the SBN and varies seasonally (Soma et al., 2003; Wacker, 2019). Altogether, these data suggest that seasonal changes in neuroestrogen synthesis contribute to seasonal changes in territorial aggression in song sparrows (Jalabert et al., 2018; Quintana et al., 2021). To measure estrogens in specific regions of the SBN, a highly specific and sensitive method is required.

Estrogens can be measured by liquid chromatography tandem mass spectrometry (LC-MS/MS), a highly specific and sensitive technique that allows for simultaneous measurement of multiple analytes. Immunoassays can suffer from cross-reactivity with structurally similar steroids (Faqehi et al., 2016). Estrogen measurement with mass spectrometry can be impeded by poor ionization efficiency, but this problem can be ameliorated by derivatization, which adds an easily ionized group or charged moiety to the analyte of interest (Faqehi et al., 2016; Denver et al., 2019b). Dansyl chloride is a commonly used derivatization reagent for estrogens. However, with dansyl chloride, the product ions are not analyte-specific because they are solely produced from the dansyl chloride moiety (Xu and Spink, 2008; Li and Franke, 2015). In addition, the sensitivity increase with dansyl chloride is not sufficient to measure estrogens in microdissected brain (C. Jalabert and K. K. Soma, unpublished results). In contrast, 1,2-dimethylimidazole-5-sulfonyl-chloride (DMIS) derivatization is estrogen-specific, generates analyte-specific product ions, produces lower background values, and yields greater sensitivity in measuring 17β-E_2_ and estrone (E_1_).

**Figure 1.**
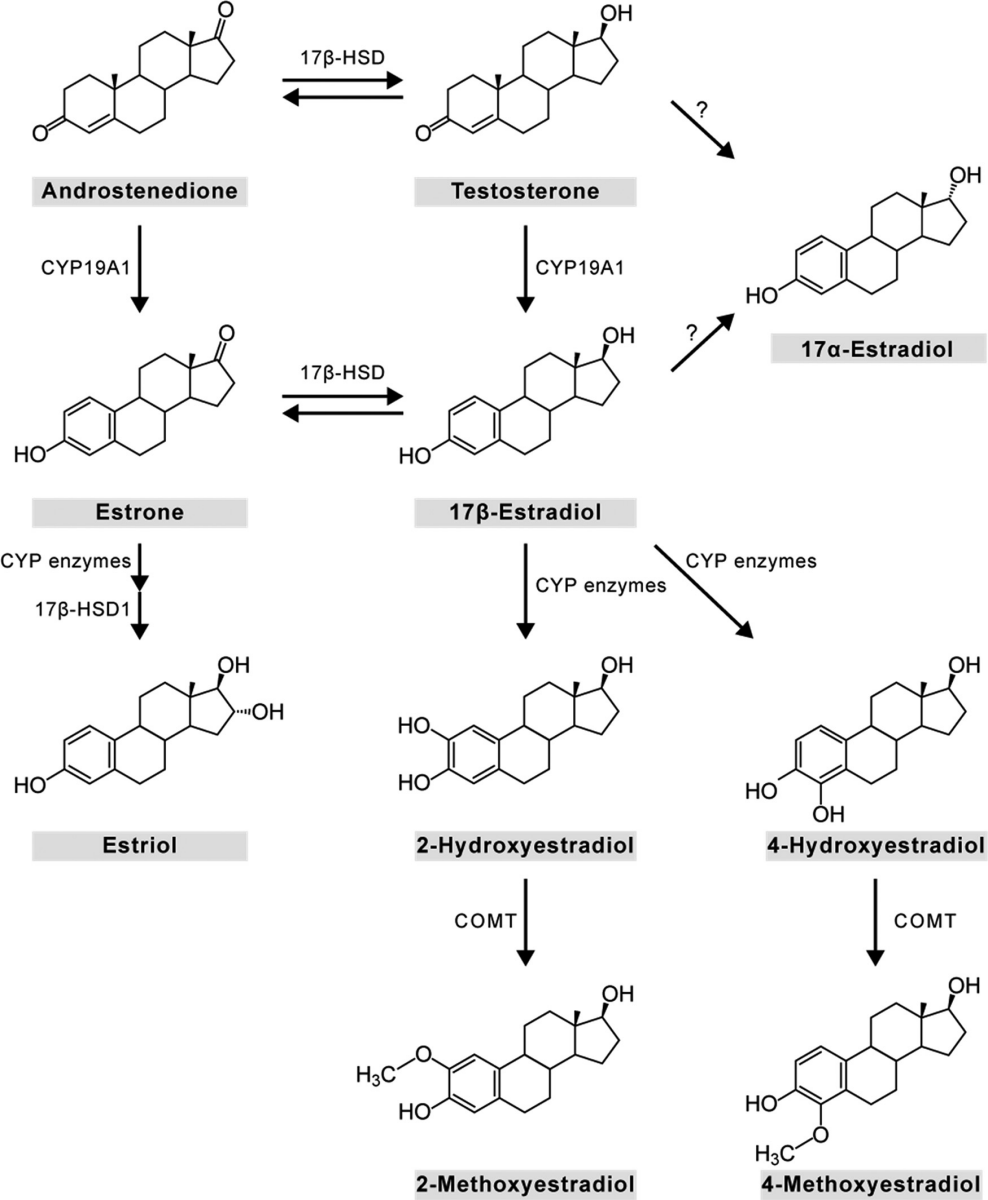
The panel of estrogens that are measured in this study. Chemical structures of E_1_, 17β-E_2_, 17α-E_2_, E_3_, 2OH-E_2_, 4OH-E_2_, 2Me-E_2_, and 4Me-E_2_. Similarly, E_1_ can be hydroxylated at the two or four positions by CYP1A1 and CYP1B1, respectively, and then methylated by the COMT enzyme to produce 2-methoxyestrone and 4-methoxyestrone. E_1_, estrone; 17β-E_2_, 17β-estradiol; 17α-E_2_, 17α-estradiol; E_3_, estriol; 2OH-E_2_, 2-hydroxyestradiol; 4OH-E_2_, 4-hydroxyestradiol; 2Me-E_2_, 2-methoxyestradiol; 4Me-E_2_; 4-methoxyestradiol. *Figure Contributions:* Melody Salehzadeh created the illustration.

Here, we developed a method to measure several estrogens simultaneously with high specificity and sensitivity, in microdissected brain regions. We derivatized estrogens with DMIS. Using LC-MS/MS, we initially aimed to measure a panel of eight estrogens: E_1_, 17β-E_2_, 17α-estradiol (17α-E_2_), estriol (E_3_), 2-hydroxyestradiol (2OH-E_2_), 4-hydroxyestradiol (4OH-E_2_), 2-methoxyestradiol (2Me-E_2_), and 4-methoxyestradiol (4Me-E_2_; Fig. 1). The method was successfully validated for E_1_, 17β-E_2_, 17α-E_2_, and E_3_ in whole blood (hereafter “blood”), plasma, and brain of song sparrows. In contrast, the method was not sufficient for quantitation of 2OH-E_2_, 4OH-E_2_, 2Me-E_2_, and 4Me-E_2_, primarily because of matrix effects. To achieve high spatial resolution, we microdissected 11 regions. We then examined seasonal changes of estrogens in blood, plasma, and microdissected brain regions in free-living male song sparrows.

## Materials and Methods

### Field procedures

Song sparrows are widespread and abundant throughout North America (especially near Vancouver) and their conservation status is of least concern, according to the IUCN red list. Free-living adult male song sparrows were captured in the nonbreeding season (October 26 to November 8, 2018, *n* = 11) and breeding season (April 9 to April 24, 2019, *n* = 10). Another four animals were captured for method validation. Subjects were captured near Vancouver using a mist net and conspecific song playback for a maximum of 5 min (breeding: 1.6 ± 0.5 min, nonbreeding: 1.6 ± 0.6 min; *p* = 0.51), to avoid effects of song playback on steroid levels. Immediately after capture, the subject was rapidly and deeply anesthetized with isoflurane and then rapidly decapitated. There was a maximum of 3 min between capture and euthanasia (breeding: 2.6 ± 0.2 min, nonbreeding: 2.2 ± 0.3 min; *p* = 0.23), to avoid effects of handling on steroid levels. The brain was immediately collected and snap frozen on powdered dry ice. Trunk blood was collected in heparinized microhematocrit tubes (Fisher Scientific) that were kept on ice packs until return to the laboratory within 5 h.

Once in the laboratory, blood was divided into two aliquots. One half of the blood sample was frozen. The other half of the blood sample was centrifuged, and then plasma was collected and frozen. All samples were stored at –70°C until steroids were extracted. Blood and plasma samples were used to measure circulating levels of estrogens. Plasma overestimates circulating steroid levels, and therefore blood was used as a more accurate estimate of circulating steroid levels and to compare to brain steroid levels (Taves et al., 2010, 2011, 2015).

All procedures were in compliance with the Canadian Council on Animal Care and protocols were approved by the Canadian Wildlife Service and the University of British Columbia Animal Care Committee.

### Brain microdissection

**Figure 2.**
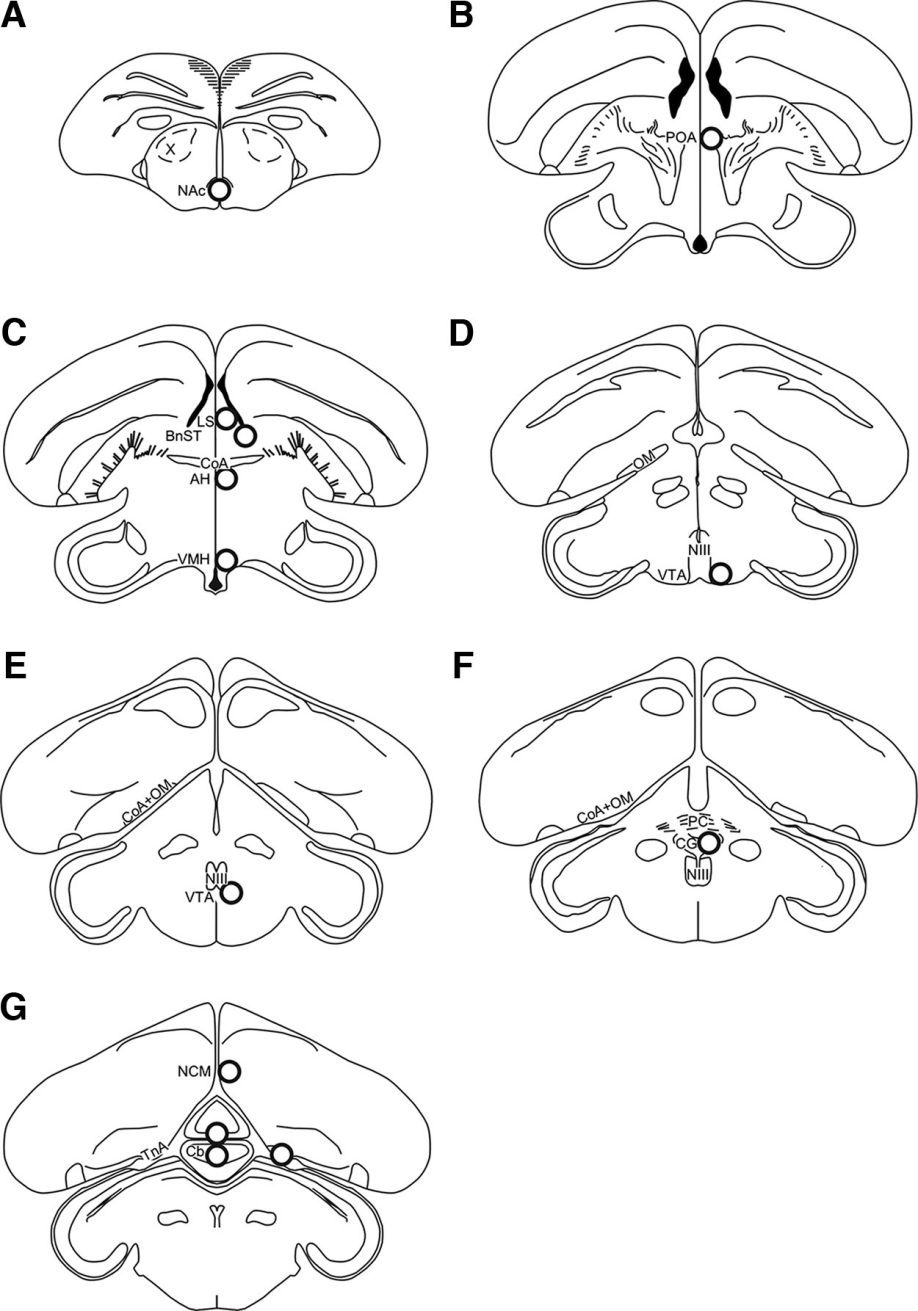
Representation of punch locations in brain slices in the coronal sections. (***A-G***) Sections are shown in a rostral to caudal order. Punches were 1-mm diameter and are shown to scale. Diagrams were adapted from the zebra finch atlas (Nixdorf-Bergweiler and Bischof, 2007), NAc, nucleus accumbens; POA, preoptic area; AH, anterior hypothalamus; LS, lateral septum; BnST, bed nucleus of the stria terminalis; VMH, ventromedial hypothalamus; VTA, ventral tegmental area; CG, central gray; NCM, caudomedial nidopallium; TnA, nucleus taeniae of the amygdala; Cb, cerebellum. Adapted from Jalabert et al. (2021). *Figure Contributions:* Melody Salehzadeh created the illustration.

To microdissect brain tissue, the Palkovits punch technique (Palkovits, 1983) was used as before (Charlier et al., 2011; Heimovics et al., 2016; Jalabert et al., 2021). Tissue was collected from 11 brain regions (Fig. 2), including those in the SBN: nucleus accumbens (NAc), preoptic area (POA), anterior hypothalamus (AH), lateral septum (LS), bed nucleus of the stria terminalis (BnST), ventromedial hypothalamus (VMH), ventral tegmental area (VTA), central gray (CG), caudomedial nidopallium (NCM), nucleus taeniae of the amygdala (TnA; homolog of the mammalian medial amygdala), and cerebellum (Cb). Brains were sectioned in the coronal plane at 300 μm using a MicroHM525 cryostatic microtome (Thermo Fisher Scientific Inc.) at −12°C while using a plane of sectioning that closely matched a zebra finch brain atlas (Nixdorf-Bergweiler and Bischof, 2007). Coronal sections were then mounted onto Superfrost Plus microscope slides (Thermo Fisher Scientific) and examined to identify brain regions of interest and major neuroanatomical landmarks.

Frozen sections were microdissected using a stainless-steel biopsy punch tool (Integra Miltex biopsy punch tool, 1-mm diameter, tissue wet weight 0.245 mg per punch). One punch (centered at the midline for one section) was collected containing the NAc, ventral to the area X. Four punches (two per side for two serial sections) were collected containing the POA in two sections caudal to the last section containing the tractus septopalliomesencephalicus and rostral to the AH. Four punches (two per side for two serial sections) were collected for the AH, immediately caudal to POA sections and ventral to the anterior commissure (CoA). Four punches (two per side for two serial sections) were collected for the LS and BnST, dorsal to the CoA. The LS was collected medial to the lateral ventricles, and the BnST was collected at the tip of each lateral ventricle. Four punches (two per side for two serial sections) containing VMH were collected ventral to the AH. Four punches (two per side for two serial sections) containing the VTA were collected ventrolateral to the oculomotor nerve. Four punches (two per side for two serial sections) containing the CG were collected ventral to the posterior commissure. Six punches (two per side for three serial sections) containing the NCM were collected starting at the last appearance of the CoA and tractus occipito-mesencephalicus path from the ventromedial telencephalon. Six punches (two per side for three serial sections) containing the TnA were collected immediately caudal to the disappearance of the CoA and tractus occipito-mesencephalicus. Six punches (two at the midline for three serial sections) containing the Cb were collected starting at its first appearance. Punches were expelled into 2-ml polypropylene tubes (Sarstedt AG & Co, 72.694.007) that each contained five zirconium ceramic oxide beads (1.4-mm diameter). Punches were then stored at −70°C until further processing.

### Reagents

**Figure 3.**
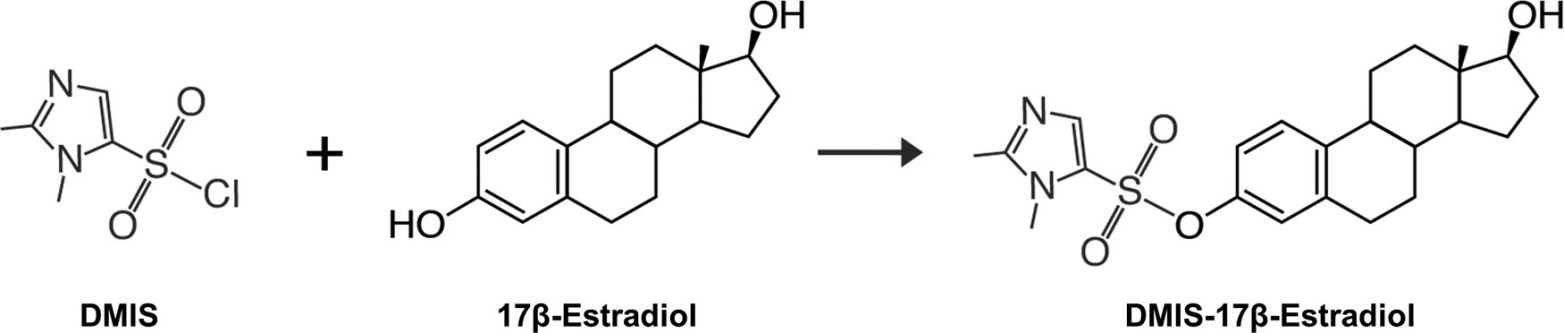
Derivatization reaction with DMIS. All estrogens of the panel react with DMIS similarly except the catechol estrogens where DMIS reacts with both hydroxyl groups of the aromatic ring. 1,2-dimethylimidazole-5-sulfonyl-chloride (DMIS).*Figure Contributions:* Melody Salehzadeh created the illustration.

High performance liquid chromatography (HPLC)-grade acetone, acetonitrile, hexane, and methanol were from Fisher Chemical. Here, we used DMIS for derivatization (Fig. 3). Note that we did not use the isomer 1,2-dimethylimidazole-4-sulfonyl chloride, which has been used for estrogen derivatization, but the fragmentation is not analyte-specific (Xu and Spink, 2007). Dry DMIS (Apollo Scientific, lot #AS478881, CAS #849351-92-4) was stored at 4°C under nitrogen gas and protected from light and moisture. Then, dry DMIS was aliquoted and stored at 4°C (protected from light and moisture but not under nitrogen gas) for up to 12 months (time of storage did not affect dry DMIS stability). Acetone was added to individual aliquots of DMIS on the day of derivatization to prepare fresh DMIS solution at 1 mg/ml. Sodium bicarbonate buffer (50 mm, pH 10.5) was prepared in Milli-Q water.

Stock solutions were prepared in HPLC-grade methanol. Certified reference standards of E_1_, 17β-E_2_, and E_3_ were obtained from Cerilliant. 17α-E_2_, 2Me-E_2_, 4Me-E_2_, and 4OH-E_2_ were obtained from Steraloids. Calibration curves were prepared in 50% methanol. The calibration curve ranged from 0.01 to 20 pg per tube for E_1_, 17β-E_2_, 17α-E_2_, E_3_, 2Me-E_2_, and 4Me-E_2_, and from 0.1 to 200 pg per tube for 4OH-E_2_. The catechol estrogens, 2OH-E_2_ and 4OH-E_2_, displayed the same fragmentation patterns and retention times after DMIS derivatization and thus were indistinguishable using our assay. As a result, we only included 4OH-E_2_ in our calibration curve. 17α-E_2_ showed the same fragmentation pattern as 17β-E_2_ and the retention time only differed by ∼0.14 min (causing the peaks to overlap). Therefore, we included 17α-E_2_ in a separate calibration curve. Internal standard (IS) stock solution of 17β-E_2_-2,4,16,16-d4 (17β-E_2_-d4, C/D/N Isotopes, catalog #D-4318, CAS #66789-03-5) was prepared in methanol and further diluted with 50% methanol to a final working solution of 40 pg/ml.

### Steroid extraction

Steroids were extracted from brain tissue (sample amount detailed above for each brain region), blood (20 μl), and plasma (20 μl) similar to before (Jalabert et al., 2021). One milliliter of acetonitrile was added to all samples, and 50 μl (i.e., 2 pg) of IS 17β-E_2_-d4 was added to all samples except “double blanks.” Samples were then homogenized using a bead mill homogenizer (Omni International Inc.) at 4 m/s for 30 s. Samples were then centrifuged at 16,100 × *g* for 5 min, and 1 ml of supernatant was taken from each sample and placed into a borosilicate glass culture tube (12 × 75 mm) that had been cleaned with methanol. After the addition of 500 μl of hexane, tubes were vortexed and centrifuged at 3200 × *g* for 2 min. Hexane was removed and discarded, and extracts were dried at 60°C for 45 min in a vacuum centrifuge (ThermoElectron SPD111V; Thermo Fisher Scientific). Calibration curves, quality controls (QCs), blanks, and double blanks were prepared alongside samples. Underivatized standards, in which acetone was added without DMIS, were prepared in parallel to measure any underivatized estrogens and calculate derivatization reaction efficiency (94–100% for all estrogens).

### DMIS derivatization

Derivatization was based on previous studies (Keski-Rahkonen et al., 2015; Handelsman et al., 2020). Here, the protocol was slightly modified to reduce reagent evaporation. Dried extracts were immersed in an ice bath, then samples were reconstituted with 30 μl of sodium bicarbonate buffer (50 mm, pH 10.5), briefly vortexed, and 20 μl of 1 mg/ml DMIS in acetone was added. Samples were then vortexed and centrifuged at 3200 × *g* for 1 min before being transferred to glass LC-MS vial inserts placed in LC-MS vials (Agilent). Vials were capped to prevent evaporation during incubation for 15 min at 60°C. This was followed by a cooling period of 15 min at 4°C. Samples were centrifuged at 3200 × *g* for 1 min, and then stored at −20°C for no more than 24 h before steroid analysis.

### Steroid analysis by LC-MS/MS

Steroids were quantified using a Sciex 6500 Qtrap UHPLC-MS/MS system (Jalabert et al., 2021). Samples were transferred into a refrigerated autoinjector (15°C). Then, 35 μl from each sample were injected into a Nexera X2 UHPLC system (Shimadzu Corp.), passed through a KrudKatcher ULTRA HPLC In-Line Filter (Phenomenex) and then an Agilent 120 HPH C18 guard column (2.1 mm), and separated on an Agilent 120 HPH C18 column (2.1 × 50 mm; 2.7 μm; at 40°C) using 0.1 mm ammonium fluoride in MilliQ water as mobile phase A (MPA) and methanol as mobile phase B (MPB). The flow rate was 0.4 ml/min. During loading, MPB was at 10% for 1.6 min, and from 1.6 to 4 min, the gradient profile was at 42% MPB, which was ramped up to 60% MPB until 9.4 min. From 9.4 to 11.9 min, the gradient was ramped from 60% to 98% MPB until 13.4 min. Finally, a column wash was performed from 11.9 to 13.4 min at 98% MPB. The MPB was then returned to starting conditions of 10% MPB for 1.5 min. Total run time was 14.9 min. The needle was rinsed externally with 100% isopropanol before and after each sample injection.

We used two multiple reaction monitoring (MRM) transitions for each estrogen and one MRM transition for the deuterated IS (Table 1). Steroid concentrations were acquired on a Sciex 6500 Qtrap triple quadrupole tandem mass spectrometer (Sciex LLC) in positive electrospray ionization mode for all derivatized estrogens and negative electrospray ionization mode for underivatized estrogens (Table 1). All water blanks were below the lowest standard on the calibration curves.

**Table 1:** Scheduled MRM for the estrogens with DMIS and without derivatization

	Mode	Retention time (min)	Quantifier *m/z*	Qualifier *m/z*
E_1_-DMIS	ESI +	10.46	429 → 365.2	429 → 96.0
17β-E_2_-DMIS	ESI +	10.74	431.0 → 367.4	431.0 → 95.9
17α-E_2_-DMIS	ESI +	10.88	431 → 367	431 → 96.0
E_3_-DMIS	ESI +	7.95	447 → 383.3	447 → 96.1
2OH-E_2_-DMIS	ESI +	10.62	605.1→ 382.3	605.1 → 96.1
4OH-E_2_-DMIS	ESI +	10.62	605.1 → 382.3	605.1 → 96.1
2Me-E_2_-DMIS	ESI +	10.99	461 → 302	461 → 161.1
4Me-E_2_-DMIS	ESI +	10.57	461 → 161.2	461 → 283.0
17β-E_2_-d4-DMIS	ESI +	10.73	435 → 371.1	435 → 96.1
E_1_	ESI –	7.61	269 → 145	269 → 143.0
17β-E_2_	ESI –	7.7	271 → 145	271 → 143.0
17α-E_2_	ESI –	8.22	271 → 145	271 → 143.0
E_3_	ESI –	3.56	287.1 → 171	287.1 → 144.9
2Me-E_2_	ESI –	8.49	301.1 → 286	301.1 → 285.0
4Me-E_2_	ESI –	8.1	301.1 → 286	301.1 → 285.0
17β-E_2_-d4	ESI –	7.63	275 → 147	—

### Stability of IS

Deuterated IS can potentially experience hydrogen-deuterium exchange (Kwok et al., 2008; Viljanto et al., 2018), so we tested for possible alterations of 17β-E_2_-d4 caused by the derivatization procedure. We compared the mass spectra of 17β-E_2_-d4 directly from the stock solution, after sham derivatization (resuspension in buffer and acetone followed by incubation for 15 min at 60°C, without DMIS), or after derivatization (resuspension in buffer and DMIS in acetone followed by incubation for 15 min at 60°C). In addition, we tested for effects of heating on the IS by comparing the mass spectra of 17β-E_2_-d4 resuspended in buffer and acetone either incubated for 15 min at 60°C or not incubated. We also examined unlabeled 17β-E_2_ either directly from the stock solution or after derivatization (resuspension in buffer and DMIS in acetone followed by incubation for 15 min at 60°C). All samples were prepared at 10 μg/ml for the infusion at 7 μl/min using a syringe pump and all other LC–MS/MS parameters were identical to those described in the previous section.

For nonderivatized samples, we evaluated quadrupole 1 (Q1) ion of 17β-E_2_ (271 *m/z*) and 17β-E_2_-d4 (275 *m/z*), and because the IS has four deuterium atoms and possible hydrogen-deuterium exchange, we scanned the mass spectra range from 270 to 276 for other Q1 ions (*m/z*, 272, 273, and 274). We also analyzed the following MRM (*m/z*) for nonderivatized samples: 271→145, 272→145, 272→146, 273→145, 273→146, 273→147, 274→146, 274→147, and 275→147. For derivatized samples, we evaluated Q1 ion of 17β-E_2_-DMIS (431 *m/z*) and 17β-E_2_-d4-DMIS (435 *m/z*) and the mass spectra range from 430 to 436. We also analyzed the following MRM (*m/z*) for derivatized samples: 431→367, 432→368, 433→369, 434→370, and 435→371.

### Assay accuracy and precision

Assay accuracy was determined by measuring QCs containing known amounts of estrogens (0.5 and 2 pg for all estrogens, except for the catechol estrogens where 5 and 20 pg were used) in neat solution. Precision was determined from both intra-assay and interassay variation by calculating the coefficient of variation of QCs. The acceptance criteria aligned with FDA style guidelines.

### Stability of derivatized analytes

Stability of derivatized analytes was assessed by measuring a 10 pg standard of E_1_, 17β-E_2_, 17α-E_2_, E_3_, 2Me-E_2_, and 4Me-E_2_ and 20 pg of 4OH-E_2_ at different storage times and temperatures. Samples were derivatized on different days, so that all samples could be injected on the same day, to ensure that LC-MS/MS conditions were the same for all samples. One set (*n* = 3) of standards was injected immediately after derivatization. The other sets of standards were injected after 24 h at 15°C, as well as after 1, 4, 8, and 31 d at −20°C or −70°C (*n* = 3 per set).

### Matrix effects and recoveries

The protocol was validated in song sparrow brain, blood, and plasma. First, matrix effects were tested by creating pools and then performing serial dilutions (0.5, 1, 2, and 4 mg for brain tissue, and 2, 5, 10, and 20 μl for blood or plasma) to assess linearity and parallelism to the calibration curves. Second, we compared the peak areas for the IS in the three matrices and neat solution. Differences in IS peak area of <20% were considered acceptable. Third, recovery was assessed by creating a pool that was divided in two, one was spiked with a known amount of steroid and the other one was unspiked. We calculated the difference in steroid concentration between those two and compared with the spike in neat solution. Recoveries were evaluated in blood, plasma, and brain at the sample amounts described above.

### Statistical analysis

A value was considered nondetectable if it was below the lowest standard on the calibration curve. When 20% or more of the samples in a group (blood or brain region) were detectable, then the nondetectable values were estimated via quantile regression imputation of left-censored missing data using MetImp web tool (Wei et al., 2018a, b; Tobiansky et al., 2020, 2021). Data were imputed for each season and each estrogen independently, and imputed values were between 0 and the lowest standard on the calibration curve. When <20% of the samples in a group (blood or brain region) were detectable, then imputations were not performed, data were not analyzed statistically, and data are only reported in the text. To compare steroid levels in brain and blood, we assumed that 1 ml of blood weighs 1 g (Taves et al., 2011; Tobiansky et al., 2020).

Statistics were conducted using GraphPad Prism version 9.02 (GraphPad Software). When necessary, data were log transformed before analysis. Regional differences in estrogen levels were analyzed by repeated measures one-way analysis ANOVA. ANOVAs were followed by Tukey multiple comparison tests and corrected *p* values are shown. Significance criterion was set at *p* ≤ 0.05. Graphs show the mean ± SEM and are presented using the nontransformed data.

## Results

We aimed to measure a panel of eight estrogens with DMIS derivatization and succeeded in measuring four estrogens (E_1_, 17β-E_2_, 17α-E_2_, and E_3_). For 2OH-E_2_, 4OH-E_2_, 2Me-E_2_, and 4Me-E_2_, there were various problems (mentioned below).

### Specificity

As seen before, DMIS interacted exclusively with the hydroxyl group on the phenolic ring and it was not reactive with the 17-hydroxyl group of estrogens (represented in Fig. 3; Keski-Rahkonen et al., 2015; Huang et al., 2021). Further, catechol estrogens showed double derivatives, but not mono derivatives as DMIS bound to both hydroxyl groups of the A-ring (Table 1).

For E_1_, 17β-E_2_, 17α-E_2_, and E_3_, the assay showed high specificity after the optimization of the liquid chromatography and scheduled MRM transitions (Table 1).

The isomers 2OH-E_2_ and 4OH-E_2_ were indistinguishable because of their identical retention times and identical quantifier and qualifier transitions (Table 1). As a result, we only included 4OH-E_2_ in the calibration curve and QCs.

### Sensitivity

**Figure 4.**
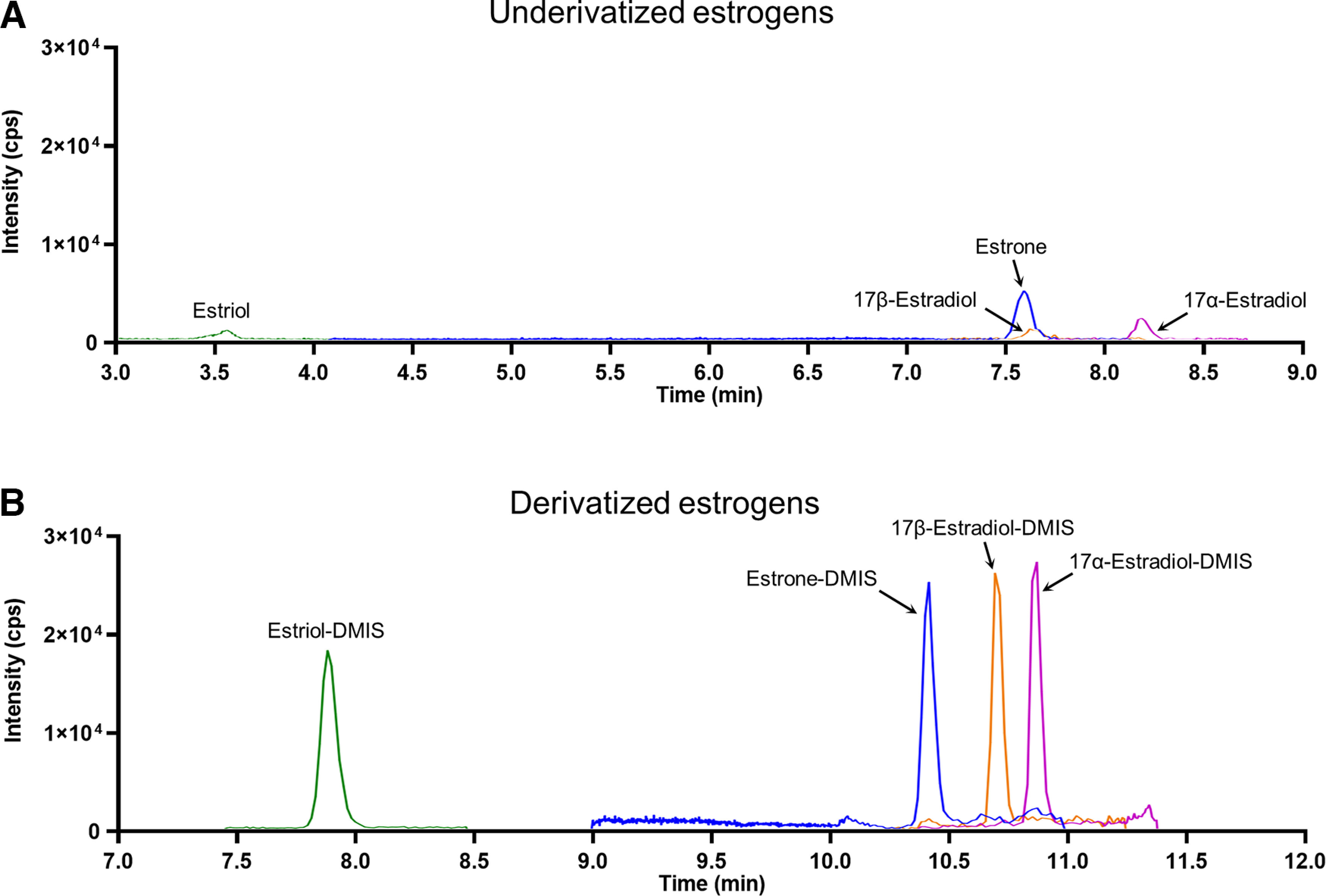
Representative chromatograms for 1 pg of each estrogen (***A***) without and (***B***) with derivatization. Intensity is expressed in cps, time is expressed in minutes.

Derivatization with DMIS greatly improved sensitivity of estrogen measurement. Using 1 pg of each estrogen in neat solution, we observed increased peak areas of analytes when derivatized with DMIS (Fig. 4*B*) compared with an assay without DMIS (Fig. 4*A*). The calibration curves were linear, even at the low range, demonstrating excellent assay sensitivity (Fig. 6).

**Figure 5.**
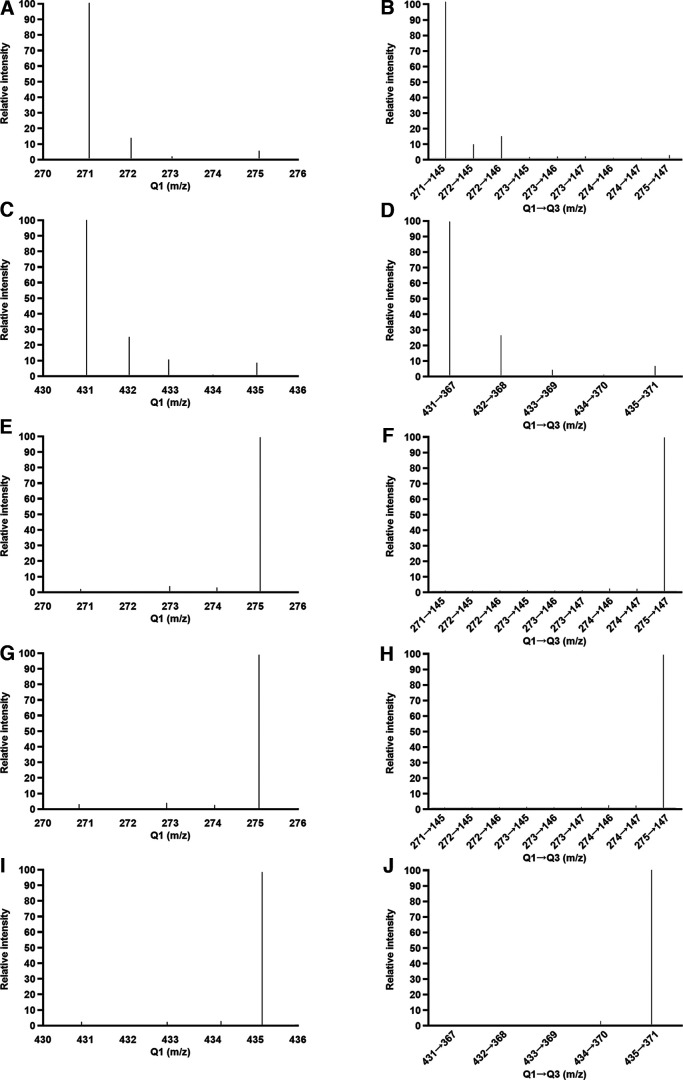
The mass spectra of Q1 scan (left panel) and the MRM (right panel) of 17β-E_2_ directly from the stock solution (***A***, ***B***) or after derivatization (***C***, ***D***) and 17β-E_2_-d4 directly from the stock solution (***E***, ***F***), after sham derivatization (***G***, ***H***), or after derivatization (***I***, ***J***). Q1, quadrupole 1; Q3, quadrupole 3; *m/z*, mass-to-charge ratio.

**Figure 6.**
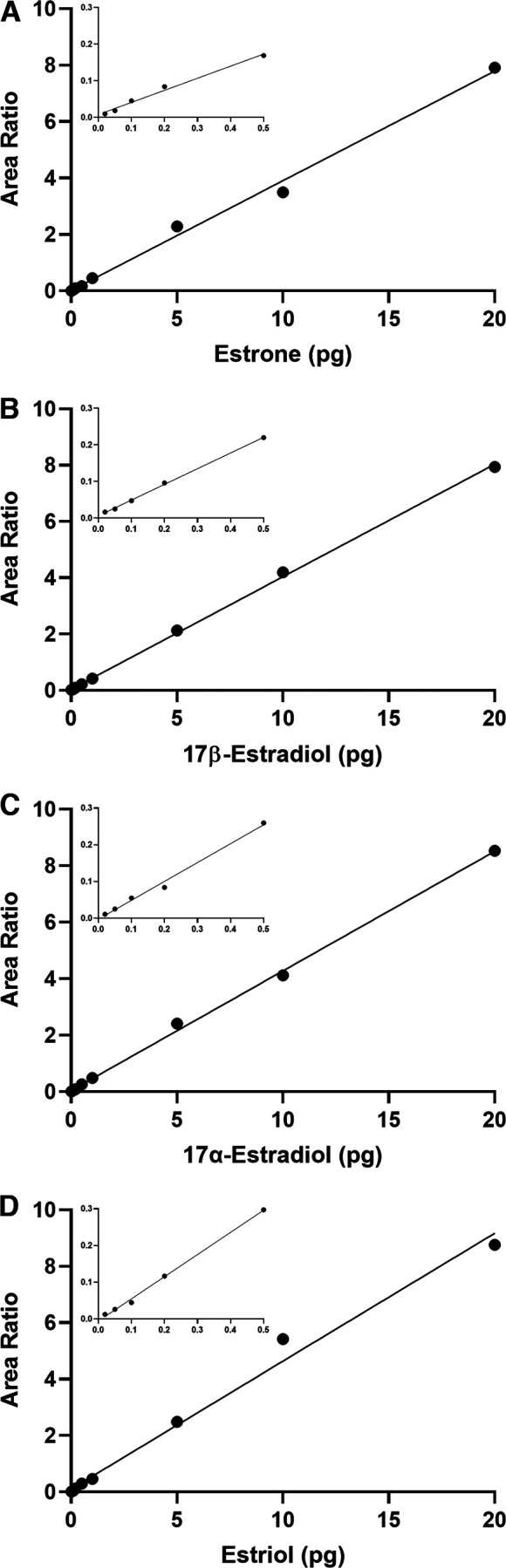
calibration curves ranging from 0.02 to 20 pg with insets displaying the lowest standards on the curve for (***A***) estrone, (***B***) 17β-estradiol, (***C***) 17α-estradiol, and (***D***) estriol. Area ratio is calculated by dividing an analyte peak area with the IS peak area in the same sample.

The lower limit of quantification (LLOQ) was enhanced by DMIS derivatization for all estrogens (Table 2). After DMIS derivatization, 17β-E_2_ and E_3_ showed a 10-fold improvement in sensitivity, and the LLOQ went from 0.2 pg/tube to 0.02 pg/tube. For 17α-E_2_ and E_1_, the LLOQ went from 0.1 to 0.02 pg/tube (Table 2).

**Table 2:** LLOQ improved by DMIS

	Underivatized (pg/tube)	Derivatized (pg/tube)
E_1_	0.1	0.02
17β-E_2_	0.2	0.02
17α-E_2_	0.1	0.02
E_3_	0.2	0.02
4OH-E_2_	-	2
2Me-E_2_	0.5	0.05
4Me-E_2_	0.5	0.2

Because of their unstable nature, 2OH-E_2_ and 4OH-E_2_ were not detected without derivatization; however following derivatization, the LLOQ was 2 pg/tube. For 2Me-E_2_ and 4Me-E_2_, the LLOQ went from 0.5 to 0.05 and 0.2 pg/tube, respectively (Table 2).

### Accuracy and precision

Accuracy and precision were measured using QCs at two amounts of estrogens in neat solution (Table 3). Accuracies were ∼100% for all estrogens at both amounts (Table 3).

**Table 3:** Accuracy, precision, and recovery in different matrices

		Low QC (0.5 pg)	High QC (2 pg)	Brain (0.5–4 mg)	Blood (1–20 μl)	Plasma (1–20 μl)
E_1_	% Recovery (% CV)	93 (14)	97 (7)	102 (5)	90 (6)	97 (5)
17β-E_2_	% Recovery (% CV)	97 (5)	101 (5)	102 (6)	94 (8)	108 (11)
17α-E_2_	% Recovery (% CV)	89 (5)	- -	- -	- -	- -
E_3_	% Recovery (% CV)	97 (6)	95 (5)	93 (3)	92 (6)	102 (7)
4OH-E_2_	% Recovery (% CV)	107 (11)	78 (16)	613 (22)	n.d.	980 (19)
2Me-E_2_	% Recovery (% CV)	96 (13)	104 (13)	214 (33)	295 (3)	291 (7)
4Me-E_2_	% Recovery (% CV)	99 (8)	92 (10)	247 (14)	200 (5)	161 (7)

Accuracy was measured by the recovery of a QC with a known concentration of estrogen. Precision was measured by the coefficient of variation (CV) of replicates. Recovery was assessed for brain, blood, and plasma by comparing unspiked samples with samples spiked with a known amount of steroid. Recovery was not assessed for 17α-E_2_ and only low QC was used for accuracy and precision so dashes are placed in those cells. n.d. is defined as nondetectable.

Precision was measured as the coefficient of variation for QC replicates at both amounts. The intra-assay variation was acceptable in all cases (Table 3). For the interassay variation, the QCs were measured across multiple assays and acceptable for E_1_ (11%), 17β-E_2_ (7%), 17α-E_2_ (8%), and E_3_ (6%).

Precision was lower for catechol and methoxy estrogens: 4OH-E_2_ (24%), 2Me-E_2_ (26%), and 4Me-E_2_ (14%).

### Stability of derivatized analytes

Stability of seven derivatized estrogens were measured at varying temperatures and durations of storage. Storage temperatures were 15°C (autosampler temperature), −20°C, and −70°C. Durations of storage were 0, 1, 4, 8, and 31 d. Analyte/IS area ratios were expressed relative to time 0 (T0), in which injection into the LC-MS/MS occurred immediately following derivatization.

All derivatized estrogens were unaffected by storage in the autosampler at 15°C for 1 d. Moreover, derivatized E_1_, 17β-E_2_, 17α-E_2_, and E_3_ were unaffected by storage up to 31 d at −20°C or −70°C (Table 5). These data indicate that the DMIS derivatives of E_1_, 17β-E_2_, 17α-E_2_, and E_3_ are stable under normal laboratory operating conditions.

In contrast, derivatized catechol and methoxy estrogens were less stable. Derivatized 4OH-E_2_, 2Me-E_2_, and 4Me-E_2_ declined after 1 d at −20°C or −70°C (Table 5). Similarly, derivatized 4OH-E_2_ decreased after 4 d at −20°C or −70°C (Table 5). Derivatized 2Me-E_2_ and 4Me-E_2_ decreased after 8 and 31 d at −20°C or −70°C (Table 5).

### Stability of IS

The Q1 and MRM of 17β-E_2_-d4 (directly from stock solution, after sham derivatization, and after DMIS derivatization) and 17β-E_2_ (directly from stock solution and after DMIS derivatization) are presented in Figure 5. The 17β-E_2_ mass spectrum is characterized by the presence of abundant 271 *m/z* deprotonated molecule (Fig. 5*A*,*B*). The mass spectra of 17β-E_2_-d4 directly from stock solution (Fig. 5*E*,*F*) and after sham derivatization (Fig. 5*G*,*H*) showed the presence of abundant 275 *m/z* molecule, indicating that deuterium loss did not occur. Derivatized 17β-E_2_ mass spectrum showed abundant 431 *m/z* (Fig. 5*C*,*D*) and, most importantly, derivatized 17β-E_2_-d4 was characterized by abundant 435 *m/z* (Fig. 5*I*,*J*). In addition, we did not detect an effect of heating 17β-E_2_-d4 (data not shown). Taken together, the data indicate the stability of the deuterated IS under the present conditions for derivatization.

### Method validation in brain matrix

First, matrix effects were assessed by creating a 60-mg pool of homogenized song sparrow forebrain tissue. This pool of brain homogenate was then spiked with estrogens and serial diluted (4, 2, 1, and 0.5 mg per tube) to evaluate linearity. The slope of each estrogen in neat solution was compared with its slope in brain tissue, to determine the extent of matrix interference. Differences in slope were measured for E_1_ (7%), 17β-E_2_ (2%), E_3_ (7%), 4OH-E_2_ (2%), 2Me-E_2_ (1%), and 4Me-E_2_ (19%) and were satisfactory (Table 4). Second, the IS peak area in brain tissue was compared with the IS peak area in neat solution and ranged from 111–118% across brain tissue amounts (0.5–4 mg). Third, recoveries were assessed by subtracting unspiked sample values from spiked sample values from the same pool and dividing by the amount of estrogen added. Recoveries were calculated across brain tissue amounts (0.5–4 mg) and were acceptable for E_1_ (102%), 17β-E_2_ (102%), and E_3_ (93%). Recoveries were high and not acceptable for 4OH-E_2_ (613%), 2Me-E_2_ (214%), and 4Me-E_2_ (247%; Table 4), suggesting matrix effects with brain tissue for 4OH-E_2_, 2Me-E_2_, and 4Me-E_2_.

**Table 4:** Assay linearity in different matrices of song sparrow

	Brain Δ slope (%)	Blood Δ slope (%)	Plasma Δ slope (%)
E_1_	7	3	0
17β-E_2_	2	1	6
E_3_	7	5	1
4OH-E_2_	2	n.d.	14
2Me-E_2_	1	3	1
4Me-E_2_	19	3	2

Difference in slope (Δ slope) was calculated by subtracting the slope of the sample with increasing amount with the slope of the standard curve in neat solution, and then dividing by the slope of the standard curve in neat solution multiplied by 100 and expressed in percentage (%). n.d. is defined as nondetectable.

### Method validation in blood matrix

First, matrix effects were assessed by creating a 266-μl pool of song sparrow blood. This pool of blood was then spiked and serial diluted (20, 10, 5, and 2 μl per tube) to evaluate linearity. The slope of each estrogen in neat solution was compared with its slope in blood. Differences in slope were measured for E_1_ (3%), 17β-E_2_ (1%), E_3_ (5%), 2Me-E_2_ (3%), and 4Me-E_2_ (3%) and were satisfactory (Table 4). However, 4OH-E_2_ was not detectable when spiked in blood (Table 4). Second, the IS peak area in blood was compared with the IS peak area in neat solution and ranged from 85–115% across blood volumes (2–20 μl). Third, recoveries were assessed by subtracting unspiked sample values from spiked sample values from the same pool and dividing by the amount of estrogen added. Recoveries were calculated across blood volumes (2–20 μl) and were acceptable for E_1_ (90%), 17β-E_2_ (94%), and E_3_ (92%). Recoveries were high and not acceptable for 2Me-E_2_ (295%) and 4Me-E_2_ (200%; Table 4), suggesting matrix effects with blood for 2Me-E_2_ and 4Me-E_2_.

### Method validation in plasma matrix

First, matrix effects were assessed by creating a 266-μl pool of song sparrow plasma. This pool of plasma was then spiked and serial diluted (20, 10, 5, and 2 μl per tube) to evaluate linearity. The slope of each estrogen in neat solution was compared with its slope in plasma. Differences in slope were measured for E_1_ (0%), 17β-E_2_ (6%), E_3_ (1%), 4OH-E_2_ (14%), 2Me-E_2_ (1%), and 4Me-E_2_ (2%) and were satisfactory (Table 4). Second, the IS peak area in plasma was compared with IS peak area in neat solution and ranged from 80% to 108% across plasma volumes (2–20 μl). Third, recoveries were assessed by subtracting unspiked sample values from spiked sample values from the same pool and dividing by the amount of estrogen added. Recoveries were calculated across plasma volumes (2–20 μl) and were acceptable for E_1_ (97%), 17β-E_2_ (108%), and E_3_ (102%). Recoveries were high and not acceptable for 4OH-E_2_ (980%), 2Me-E_2_ (291%), and 4Me-E_2_ (161%; Table 4), suggesting matrix effects with plasma for 4OH-E_2_, 2Me-E_2_, and 4Me-E_2_.

### Estrogen levels in microdissected brain regions

We examined 11 brain regions in subjects from two seasons (*n* = 10–11 subjects per season). In nonbreeding males, the only estrogen detected in the brain was 17β-E_2_ in the NCM (14.8 ± 0.9 pg/g).

**Figure 7.**
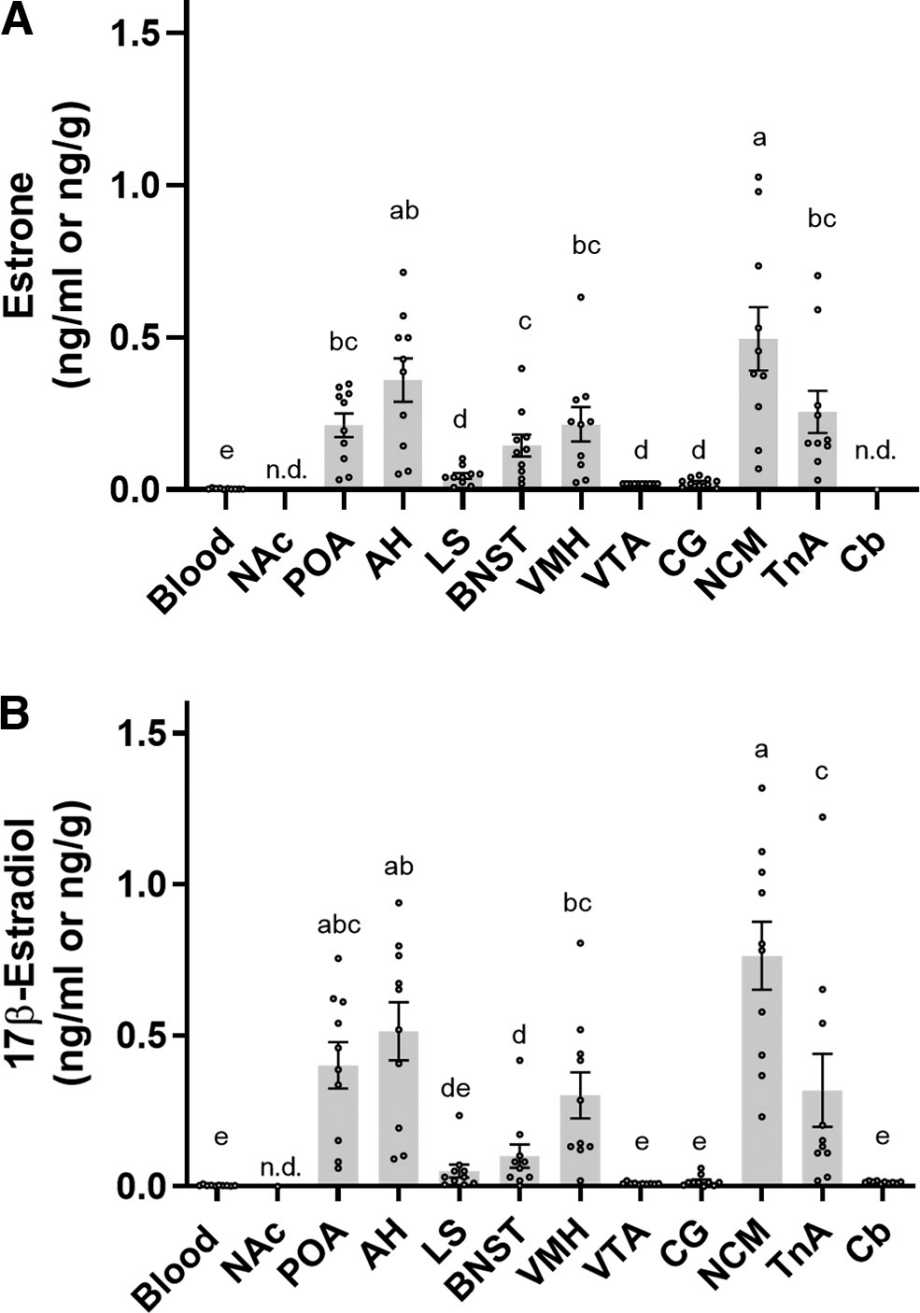
Male breeding song sparrows brain and blood levels of (***A***) estrone and (***B***) 17β-estradiol. Bar graphs represent the concentration of estrogens (ng/g brain tissue and ng/ml for blood). Values are expressed as the mean ± SEM, *n* = 10. WB, blood, NAc, nucleus accumbens; POA, preoptic area; AH, anterior hypothalamus; LS, lateral septum; BnST, bed nucleus of the stria terminalis; VMH, ventromedial hypothalamus; VTA, ventral tegmental area; CG, central gray; NCM, caudomedial nidopallium; TnA, nucleus taeniae of the amygdala; Cb, cerebellum; n.d., nondetectable.

In contrast, in breeding males, nine brain regions had detectable levels of E_1_ (Fig. 7) and ten brain regions had detectable levels of 17β-E_2_ (Fig. 7). The Cb had detectable 17β-E_2_ but not E_1_. Both E_1_ and 17β-E_2_ were nondetectable in the NAc (Fig. 7). In breeding males, E_1_ and 17β-E_2_ levels showed similar patterns across brain regions, with highest levels in NCM (Fig. 7). 17α-E_2_ and E_3_ were nondetectable in the brain of breeding males. Although there were matrix effects with brain tissue, we can very tentatively suggest that 4OH-E_2_ (LLOQ 2 ng/g), 2Me-E_2_ (LLOQ 0.05 ng/g), and 4Me-E_2_ (LLOQ 0.02 ng/g) were nondetectable in the brain of breeding males.

To compare E_1_ levels across blood and brain regions in breeding males, a one-way repeated measures ANOVA was conducted. E_1_ levels showed a significant effect of sample type (blood or brain region; *F*_(9,81)_ = 113.6, *p* < 0.0001; Fig. 7*A*). *Post hoc* comparisons revealed that E_1_ levels were higher in NCM than other brain regions (all *p* < 0.0001) except the AH. No differences in E_1_ levels were found among POA, AH, VMH, TnA; nor among POA, VMH, and BnST. Lastly, no differences were found in E_1_ levels among LS, VTA, and CG. E_1_ levels were lower in blood than in POA, AH, LS, BnST, VMH, VTA, CG, NCM, and TnA.

For 17β-E_2_ levels in breeding males, there was a significant effect of sample type (*F*_(10,90)_ = 89.10 *p* < 0.0001; Fig. 7*B*). *Post hoc* comparisons revealed that 17β-E_2_ levels were higher in NCM than other brain regions (all *p* < 0.0001) except POA and AH. No differences in 17β-E_2_ concentrations were found among VTA, CG, Cb, LS and blood; nor between TnA, VMH, and POA. 17β-E_2_ levels were lower in blood than in POA, AH, BnST, VMH, NCM, and TnA.

### Estrogen levels in circulation

In the nonbreeding season, no estrogens were detectable in the blood or plasma (*n* = 11).

In the breeding season, E_1_ was detectable in 50% of blood samples, and 17β-E_2_ was detectable in 60% of blood samples (*n* = 10). In breeding males, blood E_1_ level was 2.8 ± 0.5 pg/ml, and blood 17β-E_2_ level was 4.1 ± 0.7 pg/ml (Fig. 7). In breeding males, E_1_ and 17β-E_2_ were detectable in 70% of plasma samples (*n* = 10). Plasma E_1_ level was 3.7 ± 0.5 pg/ml, and plasma 17β-E_2_ level was 4.5 ± 0.7 pg/ml. 17α-E_2_ and E_3_ were nondetectable in the blood and plasma of breeding males. Although there were matrix effects with blood and plasma, we can very tentatively suggest that 4OH-E_2_ (LLOQ 100 pg/ml), 2Me-E_2_ (LLOQ 2.5 pg/ml), and 4Me-E_2_ (LLOQ 1 pg/ml) were nondetectable in the blood and plasma of breeding males.

## Discussion

In the present study, we developed a method to measure four estrogens (E_1_, 17β-E_2_, 17α-E_2_, and E_3_) with high specificity, sensitivity, accuracy, and precision. We also attempted to measure catechol and methoxy estrogens (2OH-E_2_, 4OH-E_2_, 2Me-E_2_, and 4Me-E_2_) but encountered various problems. We employed DMIS, an estrogen-specific derivatization reagent, with LC-MS/MS. We validated DMIS derivatization for microdissected brain tissue (1–2 mg), whereas previous work applied DMIS only with serum samples. Assay sensitivity was improved by 10-fold for some estrogens and is among the best reported in the literature. We found substantial regional and seasonal variation in neuroestrogen levels in male song sparrows. For example, the NCM, a region with high aromatase expression, has the highest E_1_ and 17β-E_2_ levels. Estrogen levels in blood are very low. Lastly, estrogen levels are lower in the nonbreeding season than in the breeding season.

### Estrogen measurement

Estrogens are present at low concentrations and similar in structure (Fig. 1); and therefore, it is challenging to measure estrogens in biological samples. Historically, estrogens have been measured with immunoassays, but these can lack the necessary specificity because of antibody cross-reaction (Faupel-Badger et al., 2010; Haisenleder et al., 2011). LC-MS/MS has higher specificity than immunoassays (Grebe and Singh, 2011; Rosner et al., 2013; Gravitte et al., 2021) and can be combined with derivatization to measure various endogenous estrogens.

Several derivatization methods are used for estrogen measurement with LC-MS/MS. Dansyl chloride is the most widely used derivatization reagent for 17β-E_2_ measurement. However, the product ion is generated from the dansyl moiety and is not specific for the analyte by mass (Xu and Spink, 2008; Li and Franke, 2015). Moreover, dansyl chloride does not provide the sensitivity required for measurement of estrogens in microdissected brain tissue (C. Jalabert and K. K. Soma, unpublished results). The reagent methyl-1-(5-fluoro-2,4-dinitrophenyl)−4-methylpiperazine (MPPZ) is useful for estrogen measurement but requires two reactions (Denver et al., 2019a). The reagent 2-fluoro-1-methylpyridinium-p-toluenesulfonate (FMP-TS) can be used to measure E_1_ and 17β-E_2_, but the derivatives decline after only 2 d of storage at −20°C (Faqehi et al., 2016). Other reagents require complex sample preparation protocols, which can be time and labor intensive (Wudy et al., 2018; Denver et al., 2019a).

DMIS has several advantages in comparison to other derivatization reagents. First, the protocol is straightforward, consisting of a single reaction with relatively mild conditions. Second, DMIS derivatization provides high specificity because product ions are analyte-specific by mass. Third, assay sensitivity is among the best reported in the literature. Fourth, DMIS reacts specifically with estrogens and allows the simultaneous measurement of nonderivatized androgens and derivatized estrogens in the same sample (Keski-Rahkonen et al., 2015; Handelsman et al., 2020).

The present study is a step forward from the pioneering work by the Handelsman group. First, DMIS was used to quantify estrogens in human and mouse serum but not in brain (Keski-Rahkonen et al., 2015; Handelsman et al., 2020). In the present study, DMIS was used for the first time to measure brain estrogens. Second, we reduced reagent evaporation during the derivatization reaction. Third, the previous studies focused and 17β-E_2_ and E_1_, and we added 17α-E_2_ and E_3_ to the panel. Fourth, we tested long-term stability of the derivatized analytes. Fifth, the previous studies used atmospheric pressure photoionization, which is relatively uncommon. This study used electrospray ionization, which is common, and makes the protocol more broadly applicable. Lastly, we tested stability of the deuterated IS and validated the use of 17β-E_2_-d4 for DMIS derivatization (see below).

### Method development

Deuterated IS are more widely available and affordable than ^13^C labeled IS. However, deuterated IS can be subject to hydrogen-deuterium exchange (Wudy et al., 2018). Here, we tested the stability of deuterated 17β-E_2_-d4 and did not observe deuterium loss (Fig. 5). Furthermore, in several brain regions (e.g., NCM, POA, VTA), the 17β-E_2_ levels observed here are similar to those observed without DMIS (Jalabert et al., 2021) indicating that DMIS derivatization yields accurate levels of 17β-E_2_. In addition, the QCs showed high accuracy and precision for E_1_, 17β-E_2_, 17α-E_2_, and E_3_ (Table 3). Low accuracy and precision in QCs often indicate hydrogen-deuterium exchange. Lastly, the same deuterated IS was used previously for derivatization with DMIS and performed well, although the IS stability was not directly assessed in these studies (Keski-Rahkonen et al., 2015; Handelsman et al., 2020).

We assessed several assay parameters. Assay specificity is key because many estrogens are similar in structure (Fig. 1). Here, E_1_, 17β-E_2_, 17α-E_2_, E_3_, 2Me-E_2_, and 4Me-E_2_ showed analyte-specific transitions patterns by mass and retention time (Table 1), whereas the 2OH-E_2_ and 4OH-E_2_ isomers were not distinguishable due retention time overlap. Assay sensitivity is also critical because estrogen amounts in blood and microdissected brain regions are extremely low. Here, DMIS derivatization improved the LLOQ for all seven estrogens (Table 2). The largest increases in sensitivity (10-fold) were observed for 17β-E_2_, 2Me-E_2_, and E_3_. This allowed measurement of E_1_ and 17β-E_2_ in regions in which we previously could not (Jalabert et al., 2021). We also assessed assay accuracy and precision, which were acceptable in all cases (Table 3). Stability of all 7 derivatized estrogens was acceptable after storage in the autosampler (15°C) for 24 h, similar to previous results on 17β-E_2_ (Keski-Rahkonen et al., 2015). Here, DMIS derivatives of E_1_, 17β-E_2_, 17α-E_2_, and E_3_ showed good long-term stability (Table 5). However, derivatized catechol and methoxy estrogens were less stable, perhaps because of oxidation (MacLusky et al., 1981). Derivative stability is an important factor but often not reported (Denver et al., 2019a). No estrogens were measured in any blanks, and some biological samples (e.g., plasma samples from nonbreeding sparrows) had nondetectable estrogen levels, indicating that this ultrasensitive assay does not produce “false positives.” Moreover, 17β-E_2_ levels in breeding NCM were very similar to previous results (without DMIS; Jalabert et al., 2021). Overall, indices of assay performance were acceptable for E_1_, 17β-E_2_, 17α-E_2_, and E_3_.

**Table 5:** Stability of estrogen DMIS derivatives

	15°C	−20°C				−70°C			
Days	1	1	4	8	31	1	4	8	31
E_1_	100	99	94	99	109	103	98	104	109
17β-E_2_	102	101	99	101	103	100	96	100	105
17α-E_2_	122	132	115	111	115	112	92	103	117
E_3_	100	97	95	99	102	98	96	101	97
4OH-E_2_	100	72	38	45	38	71	36	46	30
2Me-E_2_	100	25	79	22	28	22	75	13	17
4Me-E_2_	100	66	115	55	97	72	109	45	75

Stability was assessed in standard extracts following storage at different temperatures. Storage times were 1, 4, 8, and 31 d, and temperatures were 15°C (autosampler), −20°C, and −70°C. Values were calculated as the percentage to the area ratio measured at time 0. Time zero was defined as the time point when injection into the LC-MS/MS occurs immediately following derivatization.

Neuroestrogen measurement is also challenging because of the large amount of brain lipids that can interfere with assays (Taves et al., 2011). While many steroid extraction protocols are complex, ours is straightforward and rapid. Smaller tissue samples, as obtained by microdissection, contain less myelin and thus lower matrix effects. Estrogen measurement in large brain samples with dansyl chloride required a matrix surrogate for calibration curves, as matrix effects were present after extraction (Li and Gibbs, 2019). However, because of the limited amount of tissue obtained by microdissection (1–2 mg), there is a trade-off between reducing matrix effects and obtaining detectable quantities of estrogens. We used several techniques to assess potential matrix effects. No matrix effects were detectable with brain tissue for E_1_, 17β-E_2_, 17α-E_2_, and E_3_. In contrast, matrix effects were present with brain tissue for 4OH-E_2_, 2Me-E_2_, and 4Me-E_2_ and suggest ion enhancement (Wudy et al., 2018). Similar results were observed in blood and plasma (Tables 3, 4).

Catechol and methoxy estrogens are challenging to measure (MacLusky et al., 1981; Mesaros et al., 2014), and we faced some difficulties for their quantification. We could not distinguish between 2OH-E_2_ and 4OH-E_2_ because of co-elution. A study using the derivatization reagent MPPZ had the same issue (Denver et al., 2019c) which was partially overcome by altering the liquid chromatography (Denver et al., 2019c). Derivatization reagents can interact with either (or both) hydroxyl groups in the aromatic ring, which can hinder measurements (Denver et al., 2019b). However, DMIS produced only double derivatives, which avoided this problem. The labile nature of catechol and methoxy estrogens is shown by our stability data (Table 5). Lastly, these analytes suffered from matrix effects (Table 3). Future studies can include additional IS for catechol and methoxy estrogens to correct for matrix effects. Overall, the current assay is sufficient to determine the presence or absence of 4OH-E_2_, 2Me-E_2_, and 4Me-E_2_ in brain, blood and plasma samples but not sufficient for quantification of these analytes.

### Estrogen levels in song sparrow circulation

We examined estrogens in the circulation of wild male song sparrows. No estrogens were detectable in the circulation of nonbreeding males. In the breeding season, we observed very low concentrations of blood E_1_ and 17β-E_2_ (detectable in 50% and 60% of samples, respectively) and of plasma E_1_ and 17β-E_2_ (detectable in 70% of samples). This is consistent with previous studies in song sparrows that showed a small increase in plasma 17β-E_2_ only at the beginning of the breeding season (Soma and Wingfield, 1999). Plasma 17β-E_2_ levels were lower than those in our previous study using radioimmunoassay (Heimovics et al., 2016), probably because of the higher specificity of LC-MS/MS. 17α-E_2_ and E_3_ were not detected in blood or plasma samples from breeding males. Our data also suggest that 4OH-E_2_, 2Me-E_2_, and 4Me-E_2_ are very low in the circulation of male song sparrows. In addition, our data suggest that circulating levels of 2OH-E_2_ are very low, because we could not distinguish it from 4OH-E_2_.

### Estrogen levels in song sparrow brain regions

Estrogens are locally synthesized within the songbird brain. They can be produced either *de novo* from cholesterol or from conversion of circulating precursors (Schmidt et al., 2008; Jalabert et al., 2021). Key steroidogenic enzymes, such as 3β-hydroxysteroid dehydrogenase (3β-HSD; London et al., 2006), cytochrome P450 17α-hydroxylase/17,20-lyase (CYP17; London et al., 2003) and aromatase (Saldanha et al., 2000, 2013), are expressed in the songbird brain. In the song sparrow brain, activities of 3β-HSD and aromatase are region-specific and show seasonal changes (Soma et al., 2003; Pradhan et al., 2008, 2010). Thus, estrogen levels can differ greatly across specific brain regions. Many studies use whole brain or macro-dissection to collect large regions (e.g., forebrain or cerebral cortex), which lack spatial specificity (Li and Gibbs, 2019). In contrast, we used microdissected brain regions (1–2 mg), which allows for a simple extraction method (Grebe and Singh, 2011) and provides much greater spatial resolution.

We detected E_1_ and 17β-E_2_ in nearly all brain regions in breeding males. Overall, the present E_1_ and 17β-E_2_ brain levels match our previous data using a LC-MS/MS assay without derivatization (Jalabert et al., 2021). Importantly, the higher sensitivity of the current assay allowed us to detect estrogens in brain regions where we could not before, such as 17β-E_2_ in the BnST, CG and Cb, and both 17β-E_2_ and E_1_ in the LS and VTA. In the NAc, none of the estrogens on our panel were detectable, probably because of its small size (only one punch for NAc) and low aromatase expression (Soma et al., 2003).

Estrogen measurement in the brain is challenging, as suggested by previous studies using immunoassays. Studies from the same lab have not been able to replicate results using an immunoassay for measurement of 17β-E_2_ in zebra finch brain microdialysate, which might be because of changes in the commercial immunoassay (C. de Bournonville et al., 2020). Further, when 17β-E_2_ was measured in the same sample by both LC-MS/MS and immunoassay in quail brain microdialysate, LC-MS/MS detected far lower 17β-E_2_ concentrations than the immunoassay (M.P. de Bournonville et al., 2021), suggesting antibody cross-reactivity. When a panel of 14 estrogens was analyzed in quail brain microdialysate by LC-MS/MS, 17β-E_2_ represented <20% of total estrogens and 2OH-E_1_ levels were high (M.P. de Bournonville et al., 2021).

Estrogens in the SBN regulate a variety of social behaviors. The POA had high levels of estrogens (Fig. 7). Similarly, in male quail, estrogens are higher in the POA than in the circulation (Liere et al., 2019). Further, in the male quail POA, sexual interactions rapidly modulate aromatase activity (Cornil et al., 2005) and estrogen levels (M.P. de Bournonville et al., 2021). In wild male song sparrows, aromatase is expressed in the POA, and aromatase activity in the POA-diencephalon is higher in the breeding season than in the molt or nonbreeding season (Soma et al., 2003). Local estrogen production in the POA likely promotes sexual behavior of male sparrows. The NCM is an auditory area that contains high levels of aromatase (Saldanha et al., 2000; Soma et al., 2003) and here showed the highest levels of E_1_ and 17β-E_2_ in breeding males. In zebra finches, 17β-E_2_ levels in NCM rapidly increase in response to the presence of females or when exposed to the song of another male (Remage-Healey et al., 2008), suggesting a role for locally produced estrogens in social interactions.

There is dramatic seasonal variation in brain estrogen levels. In nonbreeding males, we only detected 17β-E_2_ in the NCM. Improved sensitivity allowed the measurement of 17β-E_2_ in the NCM of nonbreeding males, which was not possible without DMIS derivatization (Jalabert et al., 2021). Aromatase expression in the song sparrow brain is generally highest in the breeding season (Wacker et al., 2010). Consistent with this, the current data show that E_1_ and 17β-E_2_ are more abundant and widespread in the brain during the breeding season. Nevertheless, neuroestrogens promote nonbreeding aggression in male song sparrows (Soma et al., 2000b; Heimovics et al., 2015). Thus, neuroestrogen levels might be very low at baseline but increase rapidly in aggressive interactions, an idea that will be examined in a future study.

Here, we did not detect 17α-E_2_, E_3_, 4OH-E_2_, 2Me-E_2_, or 4Me-E_2_ in any brain or blood samples from wild male song sparrows. The lack of 17α-E_2_ in brain and circulation is consistent with the very low concentrations of 17α-testosterone (epitestosterone) in song sparrow plasma (Jalabert et al., 2021) as 17α-E_2_ can be synthesized from 17α-testosterone (Finkelstein et al., 1981). 17α-E_2_ is also absent in the brain and circulation of male quail (Liere et al., 2019). In quail, activity of estrogen-2-hydroxylase (CYP1A1; synthesizes 2OH-E_2_ and 2OH-E_1_) is elevated within the SBN (Balthazart et al., 1994). Catechol estrogens are then methylated by catechol *O*-methyl transferase (COMT) to produce methoxy estrogens. Here, nondetectable 4OH-E_2_ suggests that 2OH-E_2_ levels are also very low. The lack of 2Me-E_2_ is consistent with data from quail brain. In this study, males had not been challenged (no simulated territorial intrusion), which could explain why we did not detect catechol and methoxy estrogens. Future work will examine the effects of a conspecific aggressive interaction.

In conclusion, in the present study, we developed a method to measure E_1_, 17β-E_2_, 17α-E_2_, and E_3_ in brain, blood, and plasma. The derivatization improved sensitivity, making this assay among the most sensitive reported in the literature. Further, the assay showed high specificity, accuracy, and precision. Its application to the song sparrow model provides insights into the neural synthesis of estrogens in songbirds. DMIS derivatization will have wide-ranging applications for measuring estrogens in songbirds and other animal models as well as in humans.

## Synthesis

Reviewing Editor: Julie Bakker, University of Liege Decisions are customarily a result of the Reviewing Editor and the peer reviewers coming together and discussing their recommendations until a consensus is reached. When revisions are invited, a fact-based synthesis statement explaining their decision and outlining what is needed to prepare a revision will be listed below. The following reviewer(s) agreed to reveal their identity: NONE.

Synthesis: the ms has been significantly improved. However, afew minor revisions are necessary:

1) In the legend of figure 3, the authors state: “All estrogens of the panel react with DMIS similarly except the catechol estrogens where DMIS reacts with both hydroxyl groups of the aromatic ring”. This sentence is confusing. Why should DMIS only bind to C3 OH and not to C17 OH, but to both OH of the aromatic ring of the catecholestrogens Are the estradiol derivatives mono-derivatized, di-derivatized or both ?

2) Figure 5 is not really necessary.

3) In the result section, I would suggest to insert the new paragraph on the stability of the internal standards after the paragraph on the stability of derivatized analytes.
